# Health-Related Quality of Life Impairment among Patients with Different Skin Diseases in Vietnam: A Cross-Sectional Study

**DOI:** 10.3390/ijerph16030305

**Published:** 2019-01-23

**Authors:** Sau Huu Nguyen, Long Hoang Nguyen, Giang Thu Vu, Cuong Tat Nguyen, Thu Hoai Thi Le, Bach Xuan Tran, Carl A. Latkin, Cyrus S. H. Ho, Roger C. M. Ho

**Affiliations:** 1 National Hospital of Dermatology and Venereology, Hanoi 100000, Vietnam; nguyenhuusau@yahoo.com (S.H.N.); lethu2203@gmail.com (T.H.T.L.); 2 Department of Dermatology and Venereology, Hanoi Medical University, Hanoi 100000, Vietnam; 3 Center of Excellence in Behavioral Medicine, Nguyen Tat Thanh University, Ho Chi Minh City 700000, Vietnam; longnh.ph@gmail.com (L.H.N.); pcmrhcm@nus.edu.sg (R.C.M.H.); 4 Center of Excellence in Evidence-based Medicine, Nguyen Tat Thanh University, Ho Chi Minh City 700000, Vietnam; giang.coentt@gmail.com; 5 Institute for Global Health Innovations, Duy Tan University, Da Nang 550000, Vietnam; cuong.ighi@gmail.com; 6 Institute for Preventive Medicine and Public Health, Hanoi Medical University, Hanoi 100000, Vietnam; 7 Johns Hopkins Bloomberg School of Public Health, Baltimore, MD 21205, USA; carl.latkin@jhu.edu; 8 Department of Psychological Medicine, National University Hospital, Singapore 119074, Singapore; cyrushosh@gmail.com; 9 Department of Psychological Medicine, Yong Loo Lin School of Medicine, National University of Singapore, Singapore 119077, Singapore

**Keywords:** dermatology, health-related quality of life, socioeconomic status, skin disorder, Vietnam

## Abstract

Skin diseases have caused a heavy burden on the infected population worldwide. This study aimed to examine the health-related quality of life (HRQOL) among patients with different skin diseases and identify associated factors. A cross-sectional study with 430 participants was conducted at the Vietnam National Hospital of Dermatology and Venereology (NHD) from September to November 2018. The EuroQol-5 Dimensions-5 Levels (EQ-5D-5L) instrument was employed, which measures the EQ-5D index from five domains including mobility, self-care, usual activity, pain/discomfort, and anxiety/depression. Multivariate Tobit regression was adopted to determine factors that were associated with HRQOL (EQ-5D index). The rate of atopic dermatitis was the highest with 28.8%, following by contact dermatitis (17.0%) and skin fungal infections (13.0%). Regarding HRQOL, anxiety/depression was the most common health problem in patients with skin diseases (71.8%), following by pain/discomfort (63.6%). The mean EQ-5D index score was 0.73 (SD = 0.19). The lowest EQ-5D index scores were obtained for females with skin infections (mean = 0.52) and for males with psoriasis (mean = 0.59). Females had significantly lower scores compared to males (Coef. = −0.06; 95% CI = −0.11 to −0.01). Higher income and living in rural areas were also negatively correlated with the EQ-5D index. This study demonstrated the low HRQOL among patients with skin diseases in Vietnam and emphasized the vulnerability of patients with different socioeconomic statuses to their HRQOL.

## 1. Introduction

Skin diseases have been causing heavy burden all over the world regardless of age and gender, especially in tropical regions [1]. According to The Global Burden of Disease project, skin diseases were estimated to cause 41.6 million disability-adjusted life years (DALYs)—equivalent to 1.79% of the total burden of diseases [2], ranking the fourth among causes of non-fatal morbidity worldwide [3]. Dermatitis accounts for the highest burden compared to other skin diseases with 0.38% of total disease burdens globally, following by acne vulgaris (0.29%), psoriasis (0.19%), and urticaria (0.19%) [2]. Among people 65+ years old, Hahnel et al. in their systematic review indicated that the most common skin diseases were fungal infections (prevalence rate 14.3% to 64%), following by dermatitis (1% to 58.7%), xerosis (5.4% to 85.5%), and benign skin tumors (1.7% to 74.5%) [4]. 

Skin diseases cause a wide range of detrimental effects on all aspects of a patient’s life. They may suffer from physical impairment, psychological distress, and even mortality [5]. Skin diseases, in many cases, are the potentially surface presentation of more serious illnesses such as HIV/AIDS or many neglected tropical diseases [2,6]. Regarding financial burden, patients with skin diseases are also more likely to suffer high medical cost and lost work productivity [7]. Moreover, severe skin-related symptoms on the face and body can deteriorate self-esteem and self-confidence of patients, which significantly affect their involvement in social activities [8,9,10].

Due to the complicated consequences of skin diseases in patients’ lives, instead of focusing on medical outcomes alone, physicians are encouraged to pay attention to improving the overall health and well-being of these patients. One indicator that can reflect these aspects is the health-related quality of life (HRQOL) [11]. Previous studies indicated that the perception of the patient regarding their physical, mental, and emotional conditions as well as how they function socially would determine their state of wellness and burden of disease [11,12]. Moreover, the HRQOL evaluation provides a homogeneous outcome to compare the impacts of different skin diseases, as well as the effectiveness of different treatment options for each disease. There have been a number of studies attempting to measure the HRQOL of people with specific skin diseases [8,9,13,14,15]. A multicenter study was conducted in Europe in 2013 across 13 countries covering 26 categories of disease, which reported the highest HRQOL impairment in people with hidradenitis suppurativa, blistering conditions, leg ulcers, psoriasis, and eczemas [16]. However, the results of these studies varied significantly, requiring contextualized evidence for each country in order to optimize the treatment outcomes in each population.

Despite being in a region that favors the development of many skin conditions, the literature looking into prevalence of skin diseases in Vietnam as well as the HRQOL of patients have been limited. It can be argued that, especially in resource-poor settings, knowledge regarding the HRQOL of people with skin diseases would be a reference point for developing contextualized, individual-focus interventions that potentially deliver results more efficiently. Thus, this study aimed to explore the HRQOL impairments of people with different skin diseases. Moreover, we determined socioeconomic inequalities in HRQOL among these patients in order to identify the most socially vulnerable populations for the HRQOL reduction due to skin diseases in Vietnam. The results will be helpful in drawing implications for the development and implementation of appropriate care/treatment.

## 2. Materials and Methods

### 2.1. Study Design and Participants

The protocol of the study was approved by the Institutional Review Board of the Vietnam National Hospital of Dermatology and Venereology (code 855/HDDDDBVDLTU). We conducted a cross-sectional study at the outpatient clinic in the Vietnam National Hospital of Dermatology and Venereology (NHD) during the period of September–November 2018. The NHD is one of the main centers for dermatology and venereology diagnosis and treatment in Vietnam, providing services for a broad range of patients with varying illness severity. Many of the patients being treated at the NHD were previously transferred from health facilities at provincial and communal levels due to the level of severity of their conditions. At the outpatient clinic, 1500–2000 patients are examined per day.

We used a convenient sampling technique to select participants for our study among patients who were diagnosed and treated at the NHD at the time of study. Eligibility criteria for participating in our study were: (1) being 18 years old or above; (2) being able to coherently answer the questions asked by our interviewers; and (3) giving written consent expressing agreement to be involved in the study. To estimate the appropriate sample size, we used confidence level = 95%; expected mean = 0.70; expected standard deviation = 0.20 (according to a previous study in 13 European countries [16]), and absolute precision required = 0.02. The essential sample size was 385. After adding 15% of the sample size to prevent those who did not agree to participate or did not complete the interview, the final sample size was 443 patients. Finally, we successfully recruited a total of 430 participants for the study (97.1%).

### 2.2. Measurements and Instruments

Face-to-face interviews were conducted by trained medical students from Hanoi Medical University with 15–20 min per interview using a structured questionnaire in Vietnamese language. Selected participants were invited to a private room at the hospital in which interviews took place to ensure the confidentiality and quality of the answers. Data regarding the socioeconomic status of participants as well as their health status and HRQOL were collected, and are detailed below.

#### 2.2.1. Socioeconomic Characteristics and Alcohol Use

Data about gender (“What is your gender?”), age (“How old are you?”), education (“Which education level have you completed?”), marital status (What is your current marital status?”), occupation (“What is your current occupation that you spend most of the time on?”), living location (“Do you live in rural or urban area?”), and possession of health insurance (“Do you have any type of health insurance?”) were collected. We also obtained information about household monthly income (“Could you please estimate your average household monthly income from all possible sources?”), and then we divided participants into five income quintiles: lowest income, low income, middle income, high income, and highest income. All of these questions were open-ended questions, and patients were asked these questions without suggestions.

Moreover, we asked patients to report the frequency of alcohol consumption (“How often do you drink alcohol?”).

#### 2.2.2. Dermatology Conditions and Health-Related Quality of Life (HRQOL)

The information on the type and severity of dermatology diseases respondents was collected by extracting these data from patients’ medical records. The HRQOL of the patients was assessed using the EuroQOl-5 dimensions-5 levels (EQ-5D-5L) instrument. The version used in our study was translated into Vietnamese and validated in another HRQOL study in Vietnam [17,18]. The Vietnamese EQ-5D-5L has good psychometric properties with a good convergent validity, discrimination validity, and Cronbach’s alpha = 0.85 [17]. The EQ-5D-5L measured HRQOL through five domains: mobility, self-care, usual activities, pain/discomfort, and anxiety/depression. Each domain had five different respond options to choose, ranging from no problem to extreme problem/impossible to do. The combination of responses yielded 3125 unique health statuses [19]. By using a cross-walk value set developed for Vietnam, 3125 health statuses were converted into 3125 single indexes within the score range of –0.566 to 1.000 [19,20].

### 2.3. Statistical Analysis

Stata version 15.0 (Stata Corp. LP, College Station, TX, USA) was used to analyze the data. We used Mann–Whitney and Chi-squared tests to determine differences between genders. Multivariate Tobit regression, along with stepwise selection strategies (*p* < 0.2 as the threshold for selecting variables), was adopted to identify factors that were associated with the HRQOL of respondents. A *p*-value < 0.05 was considered statistically significant.

## 3. Results

Of the 430 dermatology patients in this study, the majority were female (54.7%). The mean age of participants was 36.5 (SD = 14.1) years, in which the highest proportion was the age group of 18–30 years (45.6%). Most of the patients had completed more than a high school education (66.5%) and had partners or were married (62.2%). About one third of the patients (32.1%) were freelancers, while 2.8% were unemployed. Patients were predominantly living in urban areas (62.4%) and had health insurances (83.2%). There were 44.3% of patients not drinking alcohol. Differences between male and female patients were found in age, education, marital status, occupation, and frequency of drinking alcohol (*p* < 0.05) (Table 1).

Table 2 depicts the health conditions and HRQOL of dermatology patients. The rate of atopic dermatitis was the highest with 28.8%, following by contact dermatitis (17.0%) and skin fungal infections (13.0%). Regarding HRQOL, anxiety/depression was the most common health problem in patients with skin diseases (71.8%), followed by pain/discomfort (63.6%). The mean EQ-5D index score was 0.73 (SD = 0.19). 

**Figure 1:**
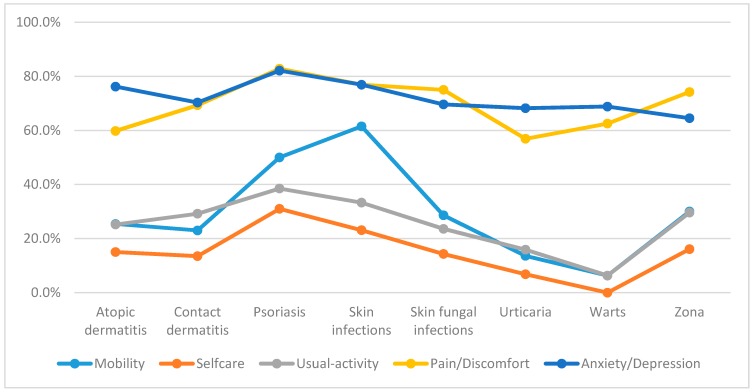
Percentage of patients reporting any problems in EuroQol-5 Dimensions-5 Levels (EQ-5D-5L) dimensions according to different dermatology disorders.

Figure 1 reveals the proportion of patients suffering problems in each EQ-5D dimension according to different dermatology diseases. The percentage of patients with skin infections having mobility problems was the highest (61.5%). Meanwhile, the rates of psoriasis patients with problems in the other four dimensions including self-care, usual activities, pain/discomfort, and anxiety/depression were the highest compared to those with other diseases (31.0%, 38.5%, 82.8%, and 82.1%, respectively).

**Figure 2:**
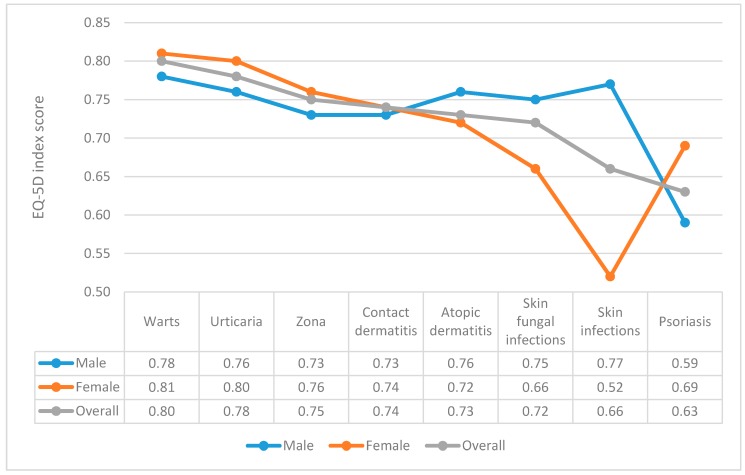
EQ-5D index according to gender and different dermatology disorders.

Regarding EQ-5D index score, warts patients had the highest score at 0.80 while psoriasis patients had the lowest score at 0.63. In terms of gender, the EQ-5D index score was the lowest in female patients with skin infections (mean = 0.52) and in male patients with psoriasis (mean = 0.59) (Figure 2).

Table 3 reveals that among dermatology patients, being female and living in rural areas were positively associated with having problems in anxiety/depression (OR = 2.35; 95% CI = 1.21–4.56 and OR = 1.94; 95% CI = 1.03–3.65, respectively). Patients drinking alcohol weekly were 3.5 times more likely to suffer anxiety/depression (OR = 3.50; 95% CI = 1.23; 9.97) than those not drinking alcohol. Those in the highest income group (OR = 3.11; 95% CI = 1.45–6.68) or living in rural areas (OR = 2.38; 95% CI = 1.40–4.03) were more likely to experience pain/discomfort compared to patients in the lowest income group or living in urban areas, respectively.

Regarding the EQ-5D index, female patients had a significantly lower score compared to males (Coef. = −0.06; 95% CI = −0.11–−0.01). Higher income (compared to the lowest income) and living in rural areas (compared to urban areas) were also negatively correlated with the EQ-5D index score. Patients who were students had markedly higher EQ-5D index scores than unemployed patients (Coef. = 0.21; 95% CI = 0.05–0.37). 

In terms of dermatology illnesses, patients having skin infections or psoriasis were more likely to suffer pain/discomfort. Patients with psoriasis also had a significantly lower EQ-5D index score compared to those not having this disease.

## 4. Discussion

The current study explored the HRQOL in patients with skin diseases as well as their socioeconomic determinants. Overall, we found a low HRQOL in both the entire sample as well as in patients with specific dermatology diseases. We also underlined that people who were female, unemployed, and living in rural areas were the populations most vulnerable to the decrement of the HRQOL if experiencing dermatology diseases. Notably, contrary to our expectation, high income was a significant predictor of poor HRQOL among dermatology patients.

In this study, we observed a low EQ-5D index with the mean value of 0.73 among dermatology patients, which was significantly lower than that in the Vietnamese general population [18], even in disadvantaged populations such as elderly, rural, or mountainous people [21,22,23]. By subgroup analysis, we confirmed the robustness of the HRQOL for each disease when compared to previous studies. For instance, the mean EQ-5D index score of urticaria patients in our study was 0.78, which was equivalent to the index score of patients experiencing moderate urticaria in three randomized clinical trials [24]. The EQ-5D index observed in our psoriasis patients (mean = 0.65) was similar to the mean score of patients with moderate-to-severe psoriasis in Europe [25], while the score in atopic dermatitis (eczema) patients (mean = 0.73) was approximately equal to this population in the United States [26]. Therefore, we believe that our results could be partly used as a vital component to compute quality-adjusted life years (QALYs), which serves as an indicator of health economic evaluation and aids in decision-making in dermatology care [27].

Our finding showed a particularly high rate of patients suffering physical and psychological impartments across dermatology diseases. The clinical impacts of dermatology diseases on depression, anxiety, and the physical health of patients have been documented [28,29,30], which were major drivers of the deterioration of the HRQOL [29,30]. Along with painful or itching conditions that patients have to suffer during the course of diseases [13,31], depression/anxiety conditions might be developed that can be explained by the onset of immunity disorders and increased proinflammatory cytokine concentrations [32]. Moreover, some previous studies indicated that patients with skin diseases might feel socially stigmatized due to their abnormal skin [33,34]. In our study, more than 70% of our respondents reported anxiety/depression, which was more severe than that of populations with other chronic conditions such as respiratory and cardiovascular diseases [29,33].

Females were found to be more greatly influenced by the dermatology illnesses compared to their male counterparts since they had a significantly lower HRQOL and a higher likelihood of having anxiety/depression. This finding was consistent with previous works [35,36,37]. The common reason for this was that physical appearance was a more sensitive issue in women than men, and dermatology disorders could develop more psychological distress among women [35]. Another study argued the differences of genetic and other molecular factors such as abnormalities in genes, metabolism, or histamin between the two genders contributed to the higher risk of depression among females [38].

Being unemployed and living in rural areas were considered significant predictors of lower HRQOL among dermatology patients. Prior studies highlighted a higher HRQOL in employed people compared to unemployed individuals due to a better life standard and earlier dermatological care access [15,18,39]. These reasons can be applied when explaining the differences in the HRQOL between rural and urban patients [9,40,41]. Interestingly, in multivariate models, we found that patients who had a higher financial status were more likely to have a poorer HRQOL and experience pain/discomfort, which was different from previous studies conducted worldwide [14,42,43]. The role of disease severity might be potential reason for these phenomena. A study in India found similar results that income was negatively correlated with HRQOL [42]. We suppose that those with a higher income also have greater concern regarding their self-esteem and appearance compared to those with a low income [44,45]. Skin diseases have been well-documented to remarkably affect the appearance and comfort of patients. In a regression model, we also found that those in the highest income group were three times more likely to suffer pain/discomfort than patients in the lowest income group. Collectively, patients with a high income possibly had a lower HRQOL than those with a low income. However, the reason for this is not clear; further studies are required to elucidate this gap.

Several suggestions could be drawn from the findings of this study. First, proper pain management and psychological counselling services should be provided to patients with skin diseases given the particularly high prevalence of pain and anxiety/depression in this population. Second, gender, living location, and occupation disparities in HRQOL suggest that more attention should be paid to female, rural, and unemployed patients, who are disadvantaged groups, in developing dermatology care services. Third, monitoring HRQOL should be performed regularly by using a short, simple instrument such as the EQ-5D-5L in order to track the progress in addressing the inequalities between different socioeconomic groups among skin disease patients.

There are a number of methodological issues that should be acknowledged in this study. First, we were unable to detect the causal relationships between socioeconomic status and HRQOL due to the nature of the cross-sectional design. Thus, further longitudinal studies are required. Second, information about HRQOL and socioeconomic status was subjectively self-reported, which could lead to measurement error. Third, using the convenient sampling technique in one hospital with a small sample size might constrain our generalizability to other dermatology populations. More studies on a large scale with different settings are warranted to confirm the effects of socioeconomic status on the HRQOL of patients with skin diseases.

## 5. Conclusions

In conclusion, this cross-section study demonstrated the low HRQOL among patients with skin diseases in Vietnam, of which warts patients had the highest HRQOL and psoriasis patients had the lowest HRQOL. This study also emphasized the impacts of socioeconomic status on the HRQOL, particularly among patients who were female, unemployed, living rural areas, and having a high household income.

## Figures and Tables

**Table 1:** Demographic characteristics of dermatology patients.

Characteristics	Male		Female		Total		*p*-Value
	*n*	%	*n*	%	*n*	%	
Total	195	45.4	235	54.7	430	100.0	
**Age group**							
18–30 years	78	40.6	113	49.8	191	45.6	0.11
31–40 years	43	22.4	45	19.8	88	21.0	
41–50 years	27	14.1	37	16.3	64	15.3	
51–60 years	24	12.5	20	8.8	44	10.5	
>60 years	20	10.4	12	5.3	32	7.6	
**Education**							
<High school	32	16.5	29	12.4	61	14.3	<0.01
High school	48	24.7	34	14.5	82	19.2	
>High school	114	58.8	171	73.1	285	66.5	
**Marital status**							
Single	63	32.5	99	42.1	162	37.8	0.04
Having partner/married	131	67.5	136	57.9	267	62.2	
**Occupation**							
Unemployed	5	2.6	7	3.0	12	2.8	<0.01
Freelancer	72	36.9	66	28.1	138	32.1	
White-collar worker	46	23.6	75	31.9	121	28.1	
Blue-collar worker	38	19.5	28	11.9	66	15.3	
Student	14	7.2	39	16.6	53	12.3	
Others	20	10.3	20	8.5	40	9.4	
**Living location**							
Urban	113	57.9	154	66.1	267	62.4	0.08
Rural	82	42.1	79	33.9	161	37.6	
**Having health insurance**							
Yes	151	81.6	186	84.5	337	83.2	0.43
No	34	18.4	34	15.5	68	16.8	
**Drinking alcohol**							
Not drinking	21	11.4	160	71.4	181	44.3	<0.01
Monthly	75	40.5	49	21.9	124	30.3	
Weekly	51	27.6	7	3.1	58	14.2	
2–3 times per week	29	15.7	5	2.2	34	8.3	
≥4 times per week	9	5.9	3	1.3	12	2.9	
	Mean	SD	Mean	SD	Mean	SD	
Age	38.1	14.6	35.1	13.6	36.5	14.1	0.04
Household monthly income (US$)	1011.9	2024.2	896.7	1204.9	948.4	1622.5	0.41

**Table 2:** Health-related quality of life (HRQOL) and health status of respondents.

Characteristics	Male		Female		Total		*p*-Value
	*n*	%	*n*	%	*n*	%	
**Dermatology diseases**							
Atopic dermatitis	44	22.6	80	34	124	28.8	<0.01
Contact dermatitis	32	16.4	41	17.4	73	17.0	0.78
Psoriasis	17	8.7	12	5.1	29	6.7	0.14
Skin infections	8	4.1	5	2.1	13	3.0	0.23
Skin fungal infections	35	17.9	21	8.9	56	13.0	<0.01
Urticaria	20	10.3	24	10.2	44	10.2	0.99
Warts	5	2.6	11	4.7	16	3.7	0.25
Zona	13	6.7	18	7.7	31	7.2	0.69
**EQ-5D-5L dimensions**							
Having problems with mobility	49	25.1	60	26.0	109	25.6	0.84
Having problems with self-care	23	11.9	34	14.5	57	13.3	0.42
Having problems with usual activity	43	22.5	60	26.2	103	24.5	0.38
Pain/discomfort	122	62.9	150	64.1	272	63.6	0.80
Anxiety/depression	126	66	179	76.5	305	71.8	0.02
**Characteristics**	**Mean**	**SD**	**Mean**	**SD**	**Mean**	**SD**	
EQ-5D index	0.74	0.19	0.722	0.19	0.73	0.19	0.39

**Table 3:** Socioeconomic factors associated with the HRQOL of dermatology patients.

Characteristics	EQ-5D Index		Having Problems with Pain/Discomfort		Having Problems with Anxiety/Depression	
	Coef.	95% CI	OR	95% CI	OR	95% CI
**Gender** (Female vs. Male)	−0.06 *	−0.11; −0.01			2.35 *	1.21; 4.56
**Education**					1.24	0.99; 1.55
**Occupation** (vs. Unemployed)						
Freelancer	0.10	−0.05; 0.25				
White-collar worker	0.14	−0.01; 0.29				
Blue-collar worker	0.12	−0.03; 0.28				
Student	0.21 *	0.05; 0.37				
Others	0.10	−0.06; 0.26				
**Household monthly income quintiles** (vs. Lowest)						
Low	−0.12 *	−0.19; −0.04	1.73	0.82; 3.64		
Middle	−0.04	−0.12; 0.04	1.26	0.60; 2.67		
High	−0.05	−0.13; 0.03	1.56	0.74; 3.29		
Highest	−0.12 *	−0.19; −0.04	3.11 *	1.45; 6.68		
**Having health insurance** (No vs. Yes)	0.05	−0.01; 0.12				
**Living location** (Rural vs. Urban)	−0.08 *	−0.14; −0.03	2.38 *	1.40; 4.03	1.94 *	1.03; 3.65
**Drinking alcohol** (vs. Not drinking)						
Monthly					1.09	0.55; 2.14
Weekly					3.50 *	1.23; 9.97
2–3 times per week					1.52	0.54; 4.34
≥4 times per week					1.46	0.32; 6.75
**Dermatology diseases**						
Contact dermatitis (Yes vs. No)			1.64	0.83; 3.22		
Skin fungal infections (Yes vs. No)			2.60 *	1.15; 5.84		
Psoriasis (Yes vs. No)	−0.15 *	−0.26; −0.05	7.25 *	1.60; 32.88	2.36	0.66; 8.41
Warts (Yes vs. No)	0.09	−0.03; 0.22				
Zona (Yes vs. No)			2.82	0.78; 10.23		

* *p* < 0.05.
